# Regulation of Granzymes A and B by High-Risk HPV: Impact on Immune Evasion and Carcinogenesis

**DOI:** 10.3390/v17020221

**Published:** 2025-02-03

**Authors:** Mashego Nathan Maleka, Zukile Mbita, Vivian Morafo

**Affiliations:** Department of Biochemistry, Microbiology and Biotechnology, School of Molecular and Life Sciences, Private Bag X 1106, Sovenga, Polokwane 0727, South Africa; 202002762@keyaka.ul.ac.za (M.N.M.); zukile.mbita@ul.ac.za (Z.M.)

**Keywords:** high-risk HPV, cancer, E6 and E7 oncoproteins, granzyme A and B, MHC-I, carcinogenesis, apoptosis, immune evasion

## Abstract

The number of new cancer cases is soaring, and currently, there are 440.5 per 100,000 new cases reported every year. A quarter of these are related to human papillomavirus (HPV) infections, particularly types 16 and 18. These include oropharyngeal, anal, vaginal, and penile cancers. A critical aspect of their oncogenic potential lies in their ability to manipulate host immune responses, facilitating immune evasion and carcinogenesis. High-risk HPVs target key immune components like granzymes A and B and MHC-I, which are crucial for the elimination of virus-infected and transformed cells, thereby weakening immune surveillance. Evidence suggests that high-risk HPVs downregulate the expression of tumor suppressors, such as p53 and pRB, and the activity of these immune components, weakening CTL and NK cell responses, thus enabling persistent infection and carcinogenesis. We discuss the implications of granzyme and MHC-I dysregulation for immune evasion, tumor progression, and potential therapeutic strategies. This review further explores the regulation of granzyme A, B, and MHC-I by high-risk HPVs, focusing on how viral oncoproteins, E6 and E7, interfere with granzyme-mediated cytotoxicity and antigen presentation. The complex interplay between high-risk HPVs, granzyme A, granzyme B, and MHC-I may provide insights into novel approaches for targeting HPV-associated cancers.

## 1. Introduction

The ubiquitous DNA virus known as the human papillomavirus (HPV) is responsible for several illnesses, including various cancers [1]. The most prominent of these is cervical cancer, which continues to rank among the main causes of cancer-related deaths linked to HPV infections globally. According to Lei et al. [2], HPV is also the cause of other cancers, such as those of the neck, mouth cavity, anal, and oropharyngeal tissues. The World Health Organization [2] reports that each year in Africa, the human papillomavirus causes over 10,702 new cases of the disease as well as 5870 fatalities. Because HPV has a strong correlation with female cancer cases, it has a significant impact on public health. Although more than 200 different strains of HPV have been identified, the strains that are most closely linked to carcinogenesis are the high-risk varieties, specifically HPV-16 and HPV-18. These high-risk strains cause recurring infections, which eventually raise the possibility of cancer. When the virus eludes the immune system and the infected cells multiply uncontrollably, cancers caused by HPV occur. The ability of the virus to obstruct cytotoxic pathways controlled by natural killer (NK) cells and cytotoxic T lymphocytes (CTLs) is a key mechanism in this immune evasion [3].

Crucial elements of the immune system, the CTLs and NK cells oversee getting rid of viral-infected and transformed cells. To achieve this, they release cytotoxic granules that contain granzymes and perforin, which cause the target cells to undergo apoptosis [4]. Granzymes A and B, which promote cell death through different but complementary mechanisms, are important players in this process. Granzyme A causes a type of cell death that is not dependent on caspase, whereas granzyme B initiates caspase-dependent apoptotic pathways. When combined, these enzymes provide a vital barrier against infections and early cancer stages. High-risk HPV strains, however, have developed cunning defenses against these immunological reactions. One of the main tactics used by HPV is the downregulation of major histocompatibility complex class I (MHC-I) molecules on the surface of infected cells [5]. For CTLs to identify and target HPV-infected cells, viral antigens must be presented to them via MHC-I molecules. HPV limits the immune system’s capacity to identify and eradicate the infected cells by decreasing or downregulating MHC-I expression, which permits the viral infection to persist within the cells.

The challenge is thus for immune cells (CTLs and NK cells) to identify HPV-infected cells whose MHC-I has been downregulated due to infection. The downregulation of MHC molecules by the human papillomavirus is by expressing viral proteins, especially E6 and E7, which are essential for carcinogenesis [6]. When functioning properly, tumor suppressor proteins such as p53 and retinoblastoma (Rb) are involved in preventing the formation of tumors. Thus, by deactivating tumor suppressor proteins like p53 and Rb, these viral proteins, E6 and E7, obstruct the host’s apoptotic machinery. The development of cancer is facilitated by this disruption, which results in unchecked cell proliferation, resistance to apoptosis, and the build-up of genetic damage. Additionally, the cytotoxic immune system response is weakened by HPV’s ability to disrupt granzyme function, which further promotes immune evasion and the advancement of malignancies linked to HPV.

## 2. Human Papilloma Viruses (HPVs)

Human papillomaviruses (HPVs) belong to the *Papillomaviridae* family, which consists of non-enveloped, double-stranded DNA viruses. They are a group of over 200 related viruses that primarily infect the skin and mucous membranes. HPVs are the most common sexually transmitted infections (STIs) worldwide, affecting epithelial cells in areas such as the cervix, anus, throat, and genital regions [7]. While many HPV infections are harmless and resolve on their own, some strains can lead to serious health issues, including genital warts, respiratory papillomatosis, and various cancers, such as cervical, anal, and oropharyngeal cancers [8]. Certain high-risk HPV strains, particularly HPV-16 and HPV-18, are known for their carcinogenic potential and are associated with most cervical cancers and other anogenital malignancies [9]. These high-risk strains can cause persistent infections and integrate into the host genome, initiating carcinogenesis through their oncogenic proteins, such as E6 and E7, which disrupt the role of critical tumor suppressor proteins [10].

The structure of human papillomavirus (HPV) is organized to enable the virus to survive in the harsh environmental conditions of host cells [11]. HPVs are small, non-enveloped viruses with a distinctive structure designed to protect their genetic material and facilitate infection of host cells. The viral particle is composed of a protein shell called the capsid, which is made up of two major proteins: L1 and L2. The L1 protein forms the outer shell of the virus and is responsible for the majority of the virus’s structure, creating an icosahedral (20-sided) symmetry [12]. This icosahedral shape provides stability to the virus and helps it survive in the external environment. The L2 protein, located internally, is involved in the assembly of the capsid and plays a role in the entry process of the virus into host cells [13]. The viral genome, which is approximately 8000 base pairs long, consists of circular double-stranded DNA. This genome contains three main regions: the early genes (E), late genes (L), and the long control region (LCR). The early genes encode proteins essential for viral replication and manipulation of the host cell, while the late genes encode the structural proteins, L1 and L2, that form the capsid [14]. The LCR regulates the transcription and replication of the viral genome.

### 2.1. Classification of HPVs

Human papillomaviruses (HPVs) are classified based on genetic similarity, oncogenic potential, and the type of tissue they infect. They belong to the family *Papillomaviridae*, which includes several genera, such as *Alpha-papillomavirus*, *Beta-papillomavirus*, *Gamma-papillomavirus*, *Mu-papillomavirus*, and *Nu-papillomavirus* [15]. Within each genus, HPVs are further classified into species and types, with each type assigned a number based on its genetic sequence. For example, *HPV-16* and *HPV-18* are among the most well-known and studied high-risk types, which are associated with cancers such as cervical, anal, and oropharyngeal cancers [16]. The classification of HPVs into low-risk and high-risk types is primarily based on their oncogenic potential. Low-risk HPV types, such as *HPV-6* and *HPV-11*, cause benign conditions like genital warts or respiratory papillomatosis, without leading to cancer [17]. In contrast, high-risk types, including *HPV-16*, *HPV-18*, *HPV-31*, and *HPV-33*, are capable of inducing persistent infections that can disrupt normal cell cycle regulation, leading to carcinogenesis [18]. These high-risk strains produce oncoproteins, such as E6 and E7, which interfere with tumor suppressor proteins like p53 and Rb, promoting the development of cancerous cells [19].

HPVs can also be classified based on the tissues they infect. Cutaneous HPVs typically infect the skin and cause conditions such as warts. These belong to genera like *Beta-papillomavirus* and *Gamma-papillomavirus* [20]. Examples include *HPV-1*, *HPV-2*, and *HPV-4*, which are linked to common warts, as well as *HPV-5* and *HPV-8*, which are associated with more serious skin conditions like epidermodysplasia verruciformis [21]. Mucosal HPVs primarily infect the mucous membranes of the genital, anal, and oral regions. Most of these belong to the *Alpha-papillomavirus* genus and can be further divided into low-risk and high-risk types [22]. For instance, *HPV-6* and *HPV-11* are low-risk types that cause genital warts, while *HPV-16* and *HPV-18* are high-risk types most often linked to cervical cancer and other anogenital malignancies. The classification of HPVs is continuously updated as new types and variants are discovered and their genetic makeup is analyzed [23]. Therefore, HPV is a highly diverse virus, with over 200 types identified, each exhibiting different tissue tropism and varying potential to cause disease, ranging from benign warts to malignancies [24].

### 2.2. Human Papillomaviruses Are Categorized into High-Risk and Low-Risk Types

Human Papillomavirus (HPV) is categorized into low-risk and high-risk types based on their potential to cause cancer. Low-risk HPV strains are typically not carcinogenic, meaning that they are not linked to cancer. Rather, they result in benign lesions that are mostly caused by HPV-6 and HPV-11, most commonly genital warts. Roughly 90% of genital warts are of these two types alone [25]. Furthermore, though uncommon, they can occasionally result in respiratory papillomatosis, a condition marked by warty growths in the respiratory tract. Low-risk strains usually do not integrate into the genome of the host cell, which reduces their ability to cause cancer. Moreover, the infections they cause are frequently transient and may go away on their own without posing a long-term threat [26].

On the other hand, if an infection with high-risk HPV persists, it may lead to a variety of cancers due to its oncogenic potential. Along with other high-risk types like HPV-31, HPV-33, and HPV-45, the main high-risk types are HPV-16 and HPV-18, which account for about 70% of cervical cancer cases globally [27]. Particularly in the cervix, persistent infection with these high-risk strains can lead to cellular alterations that eventually develop into cancer. This is due to the fact that high-risk HPVs produce two viral proteins called E6 and E7, which bind to and inactivate proteins like p53 and pRb, disrupting vital tumor-suppressor functions in the host cells [24]. Normally, these host proteins regulate cell division and prevent tumors. Nevertheless, when E6 and E7 block them, uncontrolled proliferation of cells is encouraged, which may result in cancers in the cervix, anus, genital regions, and throat [28]. However, there are more HPV strains that are implicated in the development of other diseases and cancers, and these are tabulated in Table 1. Additionally, the table shows the percentage of prevalence of these different strains.

These high-risk HPV types are managed differently. Low-risk infections frequently clear up on their own with little monitoring or treatment. On the other hand, high-risk infections are usually treated more robustly. Frequent screening is necessary to identify precancerous changes, particularly in the cervix, as early detection can stop cancer from progressing [2]. One such screening method is Pap smears. There are vaccinations against the most prevalent high-risk HPV types that offer considerable protection against cancers linked to HPV [38].

The prevalence of different types of human papillomavirus (HPV), grouped into high-risk (HR-HPV) and low-risk (LR-HPV) categories, is shown in Table 1. The high-risk HPV types, depicted at the bottom of Table 1, include types known to be associated with a higher likelihood of causing cancer, particularly cervical cancer. The low-risk types, shown at the top of Table 1, are less likely to lead to cancer and are more commonly associated with benign conditions, such as genital warts. Among the high-risk HPV types, HPV-16 stands out with the highest prevalence rate, close to 40%, making it the most common type detected in the sample. HPV-16 is well-known for its strong association with cervical and other cancers, which underscores the importance of monitoring its prevalence in populations. Following HPV-16, other high-risk types with moderate prevalence include HPV-52 and HPV-18, both of which also have links to an increased cancer risk. Additional high-risk types, such as HPV-31, HPV-58, and HPV-66, show lower but still significant prevalence, indicating they are also relatively common and may contribute to the cancer burden, though at lower rates [39].

The low-risk HPV types have generally lower prevalence rates, with HPV-54 showing the highest prevalence in this category. Other low-risk types, like HPV-6 and HPV-11, are known for causing genital warts but have minimal association with cancer development [31]. The relatively lower prevalence of these low-risk types indicates they are less commonly detected in the sample and pose a lesser cancer risk. However, they still play a role in HPV-related conditions, particularly in cases of benign warts. The data presented in Table 1 emphasize the need for targeted public health measures in terms of detection, diagnosis, and treatment. The higher prevalence of high-risk HPV types like HPV-16 and HPV-18 highlights the importance of vaccination and screening programs to prevent HPV-related cancers [40]. Understanding which HPV types are most prevalent can help guide vaccination policies and screening practices, ultimately reducing the incidence of HPV-related diseases.

### 2.3. Carcinogenic Virus

In addition to HPV, several other viruses are known to promote immune evasion and carcinogenesis through diverse mechanisms involving their viral proteins. The Epstein-Barr Virus (EBV), a herpesvirus, is associated with malignancies such as nasopharyngeal carcinoma, Burkitt’s lymphoma, and Hodgkin’s lymphoma [41]. EBV produces latent proteins, including EBNA1 (Epstein–Barr nuclear antigen 1) and LMP1 (latent membrane protein 1), which play key roles in evading immune responses and promoting cellular transformation. EBNA1 inhibits antigen processing and presentation, while LMP1 mimics a constitutively active receptor, driving cell proliferation and survival [42].

Hepatitis B Virus (HBV) and Hepatitis C Virus (HCV) are strongly linked to hepatocellular carcinoma. HBV produces the HBx protein, which disrupts tumor suppressor pathways by interacting with p53, while HCV encodes proteins such as NS3/4A protease and core protein, which impair interferon signaling and modulate host immunity, enabling persistent infection and chronic inflammation that leads to liver damage and cancer [43,44]. Human T-cell Leukemia Virus Type 1 (HTLV-1) causes adult T-cell leukemia and lymphoma, largely due to its Tax protein, which acts as a transcriptional activator that deregulates host gene expression, promotes genetic instability, and suppresses DNA repair mechanisms [45]. Another key protein, HBZ (HTLV-1 basic leucine zipper protein), enhances the proliferation and survival of infected cells while evading immune detection. Kaposi’s sarcoma-associated herpesvirus (KSHV) is linked to Kaposi’s sarcoma, primary effusion lymphoma, and multicentric Castleman disease. KSHV encodes several oncogenic proteins, such as vFLIP (viral FLICE-inhibitory protein), which inhibits apoptosis, and vGPCR (viral G protein-coupled receptor), which promotes angiogenesis and tumor growth by mimicking host signaling molecules [46].

The Merkel Cell Polyomavirus (MCPyV), associated with Merkel cell carcinoma, produces oncogenic T-antigens, including the large T-antigen and small T-antigen. These proteins drive tumorigenesis by disrupting cell cycle regulation and promoting viral genome integration into the host DNA [47]. These oncogenic viruses utilize these viral proteins to evade immune responses, promote chronic inflammation, and hijack host cellular machinery, thereby facilitating malignancy. This review focuses on HPV due to its strong association with cervical cancer.

### 2.4. The Role of Human Papillomavirus and Cytokines in Carcinogenesis

Infections with high-risk human papillomavirus are responsible for triggering carcinogenesis and evading the immune system. Mechanisms have evolved in HR-HPV infections that encourage the persistence of virus-infected cells [41]. HPV accomplishes this by inserting its viral DNA into the host’s genome, resulting in the production of two main oncoproteins, E6 and E7. These proteins interfere with critical tumor suppressor pathways within the host cells. E6 attaches to and promotes the breakdown of the p53 tumor suppressor, while E7 deactivates the retinoblastoma protein (pRb) [48]. The deactivation of these tumor suppressors leads to uncontrolled cell growth, avoidance of apoptosis, and accumulation of genetic mutations—characteristics indicative of cancer development.

Cytokines, which are small proteins essential for cell signaling, play a significant role in HPV-related carcinogenesis. Chronic HPV infection can result in ongoing inflammation, changing the cytokine profile in the affected tissue [49]. This alteration can encourage a tumor-friendly environment by suppressing the immune response and promoting an immunosuppressive microenvironment. Increased levels of cytokines such as interleukin-6 (IL-6) and tumor necrosis factor-alpha (TNF-α) are observed in HPV-associated cancers, aiding in tumor growth, angiogenesis, and metastasis [50]. Furthermore, certain cytokines can inhibit the activity of cytotoxic T cells and natural killer cells, reducing the body’s ability to eliminate HPV-infected cells and facilitating cancer progression.

### 2.5. HPV Integration and Oncogene Expression

HPV DNA integration into the host genome is a pivotal event in HPV-driven carcinogenesis, as it disrupts normal cellular functions and propels the malignant transformation of infected cells [49]. This integration leads to the sustained expression of two viral oncogenes and other replication proteins, which helps the virus to survive with the host proteins like E1, E2, E3, E4, and E5, whereas E6 and E7, which encode oncoproteins capable of subverting critical cellular regulatory mechanisms, are also involved. E6 oncoprotein primarily targets the p53 tumor suppressor protein, facilitating its degradation. p53 plays an essential role in preserving genomic stability by inducing cell cycle arrest, DNA repair, or apoptosis in response to cellular stress and DNA damage [51]. By degrading p53, E6 effectively removes these protective mechanisms, enabling the accumulation of mutations and genomic instability that contribute to cancer progression.

Similarly, the E7 oncoprotein binds to and deactivates the retinoblastoma protein (pRb), another essential tumor suppressor responsible for regulating cell cycle progression [52]. Normally, pRb functions to inhibit the cell cycle by controlling the transition from the G1 to the S phase. When E7 binds to pRb, this control is lost, allowing cells to proliferate unchecked. The combined actions of E6 and E7 create an environment in which cell division proceeds unabated, genetic mutations accumulate, and cells evade programmed cell death, setting the stage for malignancy. HPV’s oncogenic potential is further underscored by its association with various cancers, particularly in the anogenital and head and neck regions. Approximately 70% of oropharyngeal carcinomas are linked to HPV infection, with HPV-16 being the most prevalent high-risk strain involved. This link highlights the virus’s ability to persist in host cells, disrupt essential regulatory pathways, and induce oncogenic transformations that lead to cancer [53,54,55].

## 3. Cytotoxic Cytokines

Cytotoxic cytokines represent a specialized group of immune-signaling molecules that are pivotal in the body’s defense systems, primarily by coordinating immune reactions against infections, cancers, and other pathological states [56]. As members of the broader cytokine family, they govern both innate and adaptive immunity. These cytokines participate in various processes such as inflammation, cell differentiation, proliferation, survival, and programmed cell death (apoptosis). Notable members of this group include interferons (IFNs), tumor necrosis factors (TNFs), and specific interleukins (ILs), which are crucial for initiating cytotoxic actions [55]. Cytotoxic cytokines specifically aim at and eradicate infected or malignant cells by activating cell death mechanisms like apoptosis and necrosis. By fostering the elimination of abnormal or compromised cells, they help sustain tissue homeostasis and guard against disease. Nevertheless, due to their strong effects, cytotoxic cytokines require tight regulation. An imbalance in their function can result in significant tissue damage or chronic inflammation, highlighting their complexity in immune modulation [57]. Grasping the precise mechanisms behind these cytokines’ function is vital for creating targeted therapies, especially for conditions like cancer and autoimmune diseases, where immune regulation is essential for therapeutic success.

Cytotoxic cytokines encompass different subtypes, such as interferons (IFNs), tumor necrosis factors (TNFs), and interleukins, each having distinct functions and activation mechanisms [58]. Interferons (IFNs) play a vital role in defending against viruses by encouraging immune cells to identify and remove virus-infected cells and help neighboring cells develop resistance [59]. IFN-gamma (IFN-γ), an important member of this group, activates macrophages and boosts antigen presentation, aiding in pathogen recognition and destruction.

**Figure 1:**
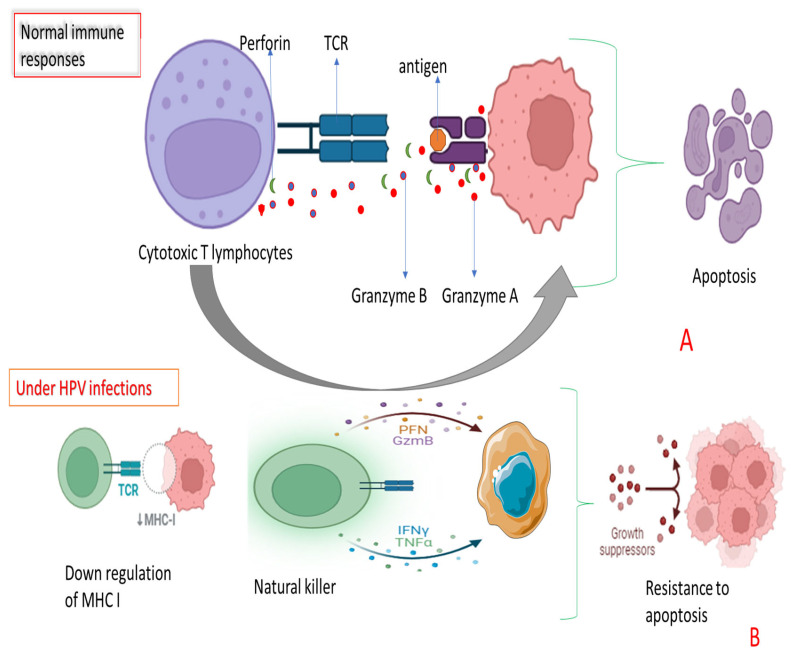
Mechanisms of immune system activation and viral immune evasion in the elimination of infected or transformed cells. (**A**) illustrates how cytokines support cytotoxic T lymphocytes (CTLs) and natural killer (NK) cells in recognizing and targeting abnormal cells (virally infected or transformed cells). (**B**) demonstrates how HPV-infected cells evade immune-mediated apoptosis. This involves downregulation of major histocompatibility complex class I (MHC-I) molecules, which prevents recognition by CTLs, allowing the infected or transformed cells to escape immune detection. However, NK cells, which do not rely on MHC-I for target recognition, can still identify and attack these cells. The figure further highlights the activity of granzymes, perforin, interferon-gamma, and interleukins in inducing apoptosis. Through these mechanisms, virally infected or transformed cancer cells undergo immune-mediated cell death. This figure was adapted from Bioreader.

Another significant group, tumor necrosis factors (TNFs), particularly TNF-alpha (TNF-α), encourages cell death in malig1nant and infected cells by triggering inflammation. TNF-α attaches to cell surface receptors, initiating signals inside the cell that may lead to apoptosis in cells with unusual growth patterns, such as cancer cells [60]. As illustrated in Figure 1 above, it shows how the cytokines fight infections of foreign pathogens. Interleukins (ILs) are also instrumental in cytotoxic processes. Although the interleukin family is broad, certain members like IL-2 and IL-12 are notably linked to boosting cytotoxic activity. IL-2, for instance, enhances T cell growth, particularly cytotoxic T lymphocytes, which are vital for spotting and eliminating infected cells. Meanwhile, IL-12 promotes the production of IFN-γ by natural killer (NK) cells and cytotoxic T cells, further strengthening immune responses [61].

These cytokines operate by triggering cytotoxic responses both directly and indirectly. Upon release, they attract immune cells like NK cells, macrophages, and cytotoxic T cells, which can directly attack and eliminate infected or malignant cells. Regulating their activity is crucial to prevent exaggerated immune responses that might cause tissue damage or autoimmune disorders [62]. Scientists are investigating ways to utilize these potent proteins in medical treatments, particularly in cancer therapy, where specifically activating cytotoxic cytokines can help in targeting tumor cells.

### 3.1. Cytotoxic T Lymphocyte and Natural Killer

Cytotoxic T lymphocytes (CTLs) and natural killer (NK) cells are key players in the immune system’s defense against infected or cancerous cells. Both types of cells are capable of inducing apoptosis (programmed cell death) in target cells, primarily through the secretion of cytotoxic molecules like granzymes and perforin [63]. Cytotoxic T lymphocytes (CTLs) are primarily activated through recognition of antigenic peptides presented by major histocompatibility complex class I (MHC-I) molecules on infected or malignant cells. This process requires the involvement of antigen-presenting cells (APCs), such as dendritic cells, which present fragments of intracellular pathogens (e.g., viruses) on MHC-I molecules. Once a naïve CD8+ T cell recognizes a specific antigen-MHC-I complex, it undergoes activation, proliferation, and differentiation into a mature CTL [64]. These activated CTLs then seek out and destroy cells presenting the same antigen on MHC-I molecules. Natural killer (NK) cells, on the other hand, do not rely on antigen recognition in the same way as CTLs. Instead, they are activated by the absence or downregulation of MHC-I molecules on target cells, which is a common feature of virally infected or cancerous cells [65]. NK cells are also stimulated by various cytokines, such as interleukin-2 (IL-2) and interferon-gamma (IFN-γ), which enhance their cytotoxic activity.

MHC-I plays a crucial role in the regulation of both CTL and NK cell activity, but their relationship with MHC-I differs significantly. CTLs require the presence of MHC-I molecules to recognize and eliminate target cells. In contrast, NK cells typically attack cells that have reduced or absent MHC-I expression [66]. Many viruses and tumors downregulate MHC-I to evade CTL recognition, which paradoxically makes them more vulnerable to NK cell-mediated killing, as NK cells detect the lack of MHC-I as a signal for destruction.

### 3.2. Role of Small Extracellular Vesicles on HPV-Infected Cells

Small extracellular vesicles (sEVs), such as exosomes, play a significant role in the progression of HPV-induced carcinogenesis by modulating cellular communication and the tumor microenvironment [67]. These nanosized vesicles, secreted by HPV-infected cells, carry bioactive molecules such as viral proteins, nucleic acids, and host-derived factors that influence nearby cells and promote malignant transformation [68].

Small extracellular vesicles derived from HPV-infected cells contribute to immune evasion by delivering immunosuppressive molecules. For instance, they may carry HPV oncoproteins E6 and E7 or viral RNA fragments, which downregulate the expression of key immune signaling molecules. By modulating antigen-presenting cells such as dendritic cells, these sEVs impair the activation of cytotoxic T lymphocytes (CTLs), allowing the infected cells to avoid immune detection [69]. Additionally, sEVs can express surface molecules like PD-L1, which binds to the PD-1 receptor on T cells, suppressing their function and facilitating immune escape. Small extracellular vesicles from HPV-infected cells play a direct role in inducing carcinogenesis by transferring oncogenic molecules to neighboring uninfected or pre-malignant cells. For example, the HPV E6 and E7 oncoproteins delivered via sEVs can inactivate tumor suppressors such as p53 and Rb in recipient cells, leading to uncontrolled cell proliferation and genomic instability. Additionally, these vesicles may carry microRNAs (e.g., miR-21, miR-155) that dysregulate cellular pathways involved in apoptosis, proliferation, and DNA repair, further promoting malignant transformation [70].

Small extracellular vesicles secreted by HPV-infected cells contribute to the creation of a tumor-supportive microenvironment. They promote angiogenesis by delivering pro-angiogenic factors such as vascular endothelial growth factor (VEGF) to endothelial cells, enhancing blood vessel formation. Moreover, they recruit regulatory T cells (Tregs) and myeloid-derived suppressor cells (MDSCs) to the tumor site, suppressing immune responses and facilitating tumor growth [71,72]. These vesicles also stimulate the production of inflammatory cytokines and chemokines, which create a chronic inflammatory state that supports cancer progression.

Small extracellular vesicles are implicated in promoting resistance to therapy and enhancing metastatic potential in HPV-associated cancers. By transferring anti-apoptotic proteins or drug-efflux transporters, sEVs help infected cells resist chemotherapy or radiotherapy [67]. Furthermore, sEVs can modify the extracellular matrix and enhance epithelial-to-mesenchymal transition (EMT) in recipient cells, enabling cancer cells to invade surrounding tissues and metastasize to distant sites. Small extracellular vesicles are critical mediators in HPV-induced carcinogenesis [67,73]. They facilitate immune evasion, promote malignant transformation, create a supportive tumor microenvironment, and contribute to therapy resistance and metastasis. Targeting sEV-mediated pathways could provide novel therapeutic strategies to combat HPV-associated cancers [74].

### 3.3. Role of Cytotoxic Cytokines in HPV Infections

Human papillomavirus infection initiates an immune response, with cytokines influencing whether the infection persists or is cleared [73]. Persistent infections can lead to cervical cancer or other cancer types. Cells infected with high-risk HPV may continue despite the immune response, progressing through the cell cycle due to viral protein production [49]. The viral DNA produces proteins necessary for their survival with host cells. In high-risk HPV infections, increased levels of proinflammatory cytokines such as IL-1α, IL-12, and TNF-α indicate a strong immune reaction attempting to eradicate the virus. These cytokines activate immune cells, including cytotoxic T-cells and natural killer (NK) cells, which specifically aim to destroy HPV-infected cells [75]. Nevertheless, sometimes HPV persists, suggesting that the immune response might not entirely succeed in eradicating the virus [76,77].

An imbalance between type 1 and type 2 cytokines is often seen in ongoing HPV infections. Type 1 cytokines, including IL-2 and IFN-γ, support a cytotoxic immune response, whereas an increase in type 2 cytokines such as IL-4 and IL-10 often appears in more advanced infection stages [78]. This imbalance diminishes the immune system’s ability to target the virus, creating conditions that enable HPV to evade immune detection and continue infecting cells. This dysfunction is associated with the development of cervical intraepithelial neoplasia (CIN) and eventually cervical cancer [79].

HPV has evolved to evade the immune system by adjusting cytokine production, challenging the body’s efforts to mount an effective response. By decreasing pro-inflammatory and cytotoxic cytokines like IFN-γ and increasing anti-inflammatory cytokines such as IL-10, HPV can evade immune detection and persist in the host. This cytokine imbalance disrupts the proper activation of immune cells, facilitating the progression of HPV-related precancerous lesions and eventually cancer [80,81].

Strategies that either stimulate cytokines like IL-12 or IFN-γ to boost immune activation or inhibit IL-10 to counteract immune evasion could offer therapeutic strategies for eliminating persistent HPV infections. However, it is crucial to precisely control these therapies because excessive or prolonged inflammation induced by cytotoxic cytokines might result in chronic conditions that promote cancer development [78]. Ultimately, cytotoxic cytokines are vital in the immune system’s fight against HPV infections, affecting both the virus’s persistence and cervical cancer development. A thorough understanding of cytokine regulation and the immune landscape is essential for creating treatments that effectively clear the virus without triggering harmful inflammatory reactions.

### 3.4. Granzymes

Granzymes are a family of serine proteases that play a crucial role in the immune system’s ability to induce apoptosis (programmed cell death) in infected, cancerous, or otherwise damaged cells [82]. These enzymes are produced and secreted by cytotoxic immune cells such as cytotoxic T lymphocytes (CTLs) and natural killer (NK) cells, which act as key defenders against intracellular pathogens, including viruses, and abnormal cells like tumors [83]. Granzymes are enzymes with a serine residue in their active site, giving them their protease activity. There are five known types of granzymes in humans, the most cell-studied of which are granzyme A and granzyme B. These granzymes are stored in the cytotoxic granules of CTLs and NK cells alongside perforin, a pore-forming protein. When these immune cells recognize a target cell, they release the contents of their granules, with perforin forming pores in the target cell membrane [84].

Perforin is a protein that plays a vital role in the immune system, particularly in defending against infections and cancer [85,86]. It is produced by cytotoxic T cells and natural killer (NK) cells, which are essential components of the body’s immune response. Perforin functions by forming pores in the membrane of target cells, such as those infected by viruses or transformed into cancerous cells [87]. Once these pores are formed, other molecules, particularly granzymes, can enter the target cell and trigger apoptosis, or programmed cell death. This mechanism allows cytotoxic T cells and NK cells to eliminate harmful cells effectively. Perforin’s action is tightly regulated to ensure that only infected or abnormal cells are targeted, preventing damage to healthy cells [88]. A deficiency or dysfunction of perforin can lead to serious conditions such as familial hemophagocytic lymphohistiocytosis (FHL), a disorder in which the immune system fails to properly eliminate infected or malignant cells, resulting in tissue damage [89]. Thus, perforin is crucial for maintaining the body’s ability to fight off infections and cancer while preserving immune balance.

Granzymes then enter the cell through these pores to execute their lethal function. Once inside the target cell, different granzymes trigger distinct biochemical pathways leading to apoptosis. Granzyme B is especially important, as it activates caspases, which are enzymes that break down key proteins in the cell and initiate the apoptotic cascade [90]. Granzyme B can also directly cleave proteins within mitochondria, leading to the release of cytochrome c, further promoting apoptosis. Granzyme A, on the other hand, induces a caspase-independent form of cell death by damaging nuclear DNA and activating alternative cell death pathways [91]. Granzymes are critical in the immune response against viral infections, tumors, and certain intracellular bacteria. By inducing apoptosis, granzymes help eliminate infected or transformed cells without causing widespread inflammation or damage to the tissues. This targeted killing is a hallmark of the immune system’s precision and efficiency.

### 3.5. Regulation of Granzymes

Granzymes are serine proteases produced by cytotoxic T lymphocytes (CTLs) and natural killer (NK) cells, crucial for inducing apoptosis in infected or malignant cells [82]. Their regulation is tightly controlled to ensure they specifically target pathological cells while avoiding damage to healthy tissues. This regulation begins at the genetic level, where transcription of granzyme genes is directed by factors like T-bet and Eomesodermin [92]. These transcription factors are activated in response to cytokines such as IL-2, IL-12, and type I interferons. Additionally, epigenetic mechanisms like chromatin remodeling and histone modifications play a role in modulating their expression. Once synthesized, granzymes are stored as inactive precursors in cytotoxic granules within CTLs and NK cells [93]. This sequestration prevents the granzymes from harming their host cells. Within the granules, granzymes are activated by the enzyme dipeptidyl peptidase I (also called cathepsin C), which removes an inhibitory propeptide. These granules are released only upon immunological synapse formation between the cytotoxic cells and their target, ensuring that granzyme release occurs specifically at the site of engagement [94]. Perforin, another granule protein, facilitates the delivery of granzymes into the target cell by creating membrane pores or aiding in endosomal escape.

Regulation also occurs through the action of endogenous inhibitors. Serpins, such as Serpin B9 (PI-9), neutralize granzymes within cells that need to resist cytotoxic attack, like antigen-presenting or immune-privileged cells [95]. In the extracellular environment, granzymes are rapidly degraded or inhibited by soluble factors and environmental conditions, such as pH, to prevent unintended damage to surrounding tissues. Post-translational modifications like glycosylation further regulate granzyme activity, enhancing their stability and modulating their interactions with other molecules. Cytokines and signaling pathways also play critical roles in granzyme regulation. IL-2 and IL-15 enhance granzyme production and activity, while activation of receptors such as the T-cell receptor (TCR) or NK cell-activating receptors ensures granzyme release is tightly linked to target cell recognition [96]. After their release, extracellular granzymes are quickly cleared or inactivated, minimizing the risk of collateral damage. Dysregulation of granzymes can lead to pathological conditions such as autoimmune diseases, chronic inflammation, or immune evasion by cancer cells [97,98].

### 3.6. Role of Granzymes and Perforin in Regulation of HPV-Infected Cells

**Figure 2:**
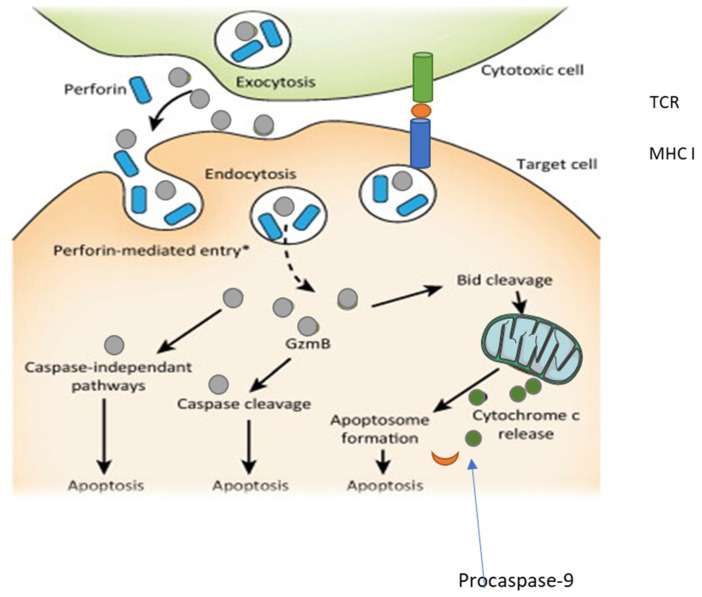
The role of major histocompatibility complex class I (MHC-I) in cytotoxic cytokine release. The figure illustrates the association between MHC-I and the release of cytotoxic cytokines, including granzyme A, granzyme B, and perforin. Immune cells, such as cytotoxic T lymphocytes (CTLs) and natural killer (NK) cells, recognise abnormal or infected cells through antigen presentation on MHC-I molecules. This recognition triggers the activation of immune cells and the subsequent release of cytotoxic molecules, granzymes A and B, along with perforin, which induce apoptosis in target cells. This diagram was adapted from Hiebert and Granville [98].

These molecules are primarily produced by cytotoxic T lymphocytes (CTLs) and natural killer (NK) cells, which are essential for recognizing and eliminating virus-infected cells. Cytotoxic T lymphocytes (CTLs) and natural killer (NK) cells, recognise abnormal or infected cells through antigen presentation on major histocompatibility complex molecules leading to elimination of infected or target cells (Figure 2). In the context of HPV infection, which can lead to cervical and other cancers, the regulation of infected cells by granzymes and perforin is an important mechanism to prevent the persistence of the virus and its progression to malignancy [96].

When CTLs or NK cells recognize an HPV-infected cell, they release perforin, which forms transmembrane pores in the infected cell’s membrane [99]. This creates a gateway for granzymes, which are serine proteases, to enter the cell. Once inside, granzymes trigger apoptosis, a programmed cell death mechanism. By inducing apoptosis, granzymes ensure the elimination of infected cells before the virus can replicate and spread, effectively curbing the viral load and reducing the likelihood of long-term infection. Granzyme B is one of the most potent granzymes involved in killing HPV-infected cells. It activates caspases, a family of enzymes that play a critical role in executing apoptosis. Additionally, granzymes can degrade viral proteins and disrupt the replication process within infected cells. This not only leads to the death of the infected cell but also ensures that the virus is destroyed, thereby limiting the potential for further infection [100]. The combined actions of perforin and granzymes serve as a powerful immune response to control HPV infection and prevent the development of HPV-related cancers.

Some HPV strains have evolved mechanisms to evade immune responses, including resistance to perforin and granzyme-induced apoptosis [101]. For instance, HPV can inhibit the activation of immune cells or interfere with apoptotic pathways in infected cells, allowing the virus to persist in the host and increasing the risk of developing cancer [19].

### 3.7. The Implications of Granzymes in Carcinogenesis

Granzymes are serine proteases primarily secreted by cytotoxic T lymphocytes (CTLs) and natural killer (NK) cells as part of the immune response to eliminate infected or malignant cells. These enzymes, particularly granzyme A (GZMA) and granzyme B (GZMB), play crucial roles in inducing apoptosis in target cells [102]. However, their implications in carcinogenesis are multifaceted, as they can either suppress or promote cancer progression, depending on the context [103].

#### 3.7.1. Anti-Tumor Effects of Granzymes

Granzymes are central to the immune system’s ability to combat tumor cells. Granzyme B, for example, induces apoptosis by cleaving caspases and other intracellular substrates, disrupting cellular integrity. This mechanism is essential for eliminating cancer cells and preventing their proliferation [104]. Studies have shown that higher activity of granzymes correlates with improved immune surveillance and tumor suppression. Granzymes also play a role in modulating the tumor microenvironment by limiting the growth of cancer-supporting cells, such as fibroblasts and endothelial cells, which contribute to angiogenesis [105].

#### 3.7.2. Tumor-Promoting Roles of Granzymes

Granzymes may also have tumor-promoting effects under certain conditions [106]. For instance, chronic inflammation and an impaired immune response can lead to the dysregulated release of granzymes, which may promote tissue damage and create a microenvironment conducive to tumor growth [89]. Granzyme A has been implicated in promoting pro-inflammatory cytokine release and reactive oxygen species (ROS) generation, which can enhance DNA damage and genomic instability, contributing to carcinogenesis [97]. Additionally, some tumor cells develop resistance to granzyme-induced apoptosis by expressing inhibitors such as serpins, which can neutralize granzyme activity [107].

## 4. Carcinogenesis

### 4.1. Cell Survival Pathways

Cell survival pathways are essential for maintaining cellular homeostasis, regulating growth, and ensuring that cells can adapt to normal physiological conditions or respond to stress [108]. Under both normal and stress conditions, these pathways help cells survive by promoting pro-survival signals and suppressing apoptotic mechanisms. Stressors such as nutrient deprivation, oxidative stress, and DNA damage can activate these pathways, allowing cells to adapt and survive [109]. However, when these pathways are dysregulated, they can contribute to the development of diseases such as cancer, where cells evade apoptosis and proliferate uncontrollably [110,111]. Below, we discuss some of the key cell survival pathways, how they are activated, and their roles in maintaining cellular health and in disease, particularly cancer.

One of the most central cell survival pathways is the PI3K/AKT/mTOR pathway, which is involved in regulating growth, metabolism, and cell survival [112]. It is activated when growth factors, such as epidermal growth factor (EGF) or insulin-like growth factor (IGF), bind to their respective receptors on the cell surface. This binding triggers the activation of phosphoinositide 3-kinase (PI3K), which phosphorylates phosphatidylinositol lipids in the plasma membrane [113]. These phosphorylated lipids recruit AKT (also known as protein kinase B) to the membrane, where it becomes activated [114].

Once activated, AKT promotes cell survival by inhibiting pro-apoptotic proteins like Bad and caspase-9, preventing the initiation of apoptosis. Additionally, AKT activates mTOR (mechanistic target of rapamycin), a key regulator of cell growth and metabolism [112]. mTOR helps control protein synthesis, autophagy, and cellular energy balance, thereby supporting cell growth and survival. Dysregulation of this pathway—through overactivation of PI3K, AKT, or mTOR—is commonly observed in cancers, leading to resistance to apoptosis, uncontrolled cell growth, and therapy resistance [115]. Targeting components of this pathway with inhibitors is an ongoing therapeutic strategy in oncology.

Another crucial pathway for cell survival is the NF-κB pathway. NF-κB is a family of transcription factors involved in regulating immune response, inflammation, and cell survival. Under normal conditions, NF-κB is sequestered in the cytoplasm by inhibitory proteins known as IκBs [116]. In response to various stimuli, such as inflammatory cytokines, stress signals, or viral infections, the IκB kinase (IKK) complex becomes activated. This activation leads to the degradation of IκB, which allows NF-κB to translocate into the nucleus. In the nucleus, NF-κB activates the transcription of genes that regulate inflammation, cell survival, and proliferation [117].

NF-κB promotes cell survival by upregulating anti-apoptotic proteins such as Bcl-2 and cellular inhibitors of apoptosis (cIAPs). These proteins inhibit the activation of caspases, which are key components of the apoptotic machinery. Chronic or persistent activation of NF-κB has been linked to various cancers and chronic inflammatory conditions, as it allows cells to evade apoptosis and continue proliferating even in the presence of DNA damage or stress [97].

The MAPK/ERK pathway is also essential for promoting cell survival and proliferation. This pathway is activated by various extracellular signals, including growth factors and mitogens that bind to receptors on the cell surface [118]. The activation of these receptors triggers a cascade of phosphorylation events, beginning with MAPK kinase kinases (MAP3Ks), which activate MAPK kinases (MAP2Ks), and finally lead to the activation of MAP kinases (ERKs). ERKs, after their activation, enter the nucleus and promote the transcription of genes involved in cell survival and proliferation [119]. Additionally, ERKs inhibit pro-apoptotic factors, such as Bax, and activate survival factors like Bcl-2. Dysregulation of this pathway is frequently associated with cancer, as mutations in upstream signaling components such as Ras or Raf can result in constitutive activation of the pathway, leading to enhanced cell survival and tumorigenesis [120]. This makes the MAPK/ERK pathway a key target for cancer therapies.

The JAK/STAT pathway is another important mediator of cell survival signals, particularly in response to cytokines and growth factors. Activation of this pathway begins when Janus kinases (JAKs) are activated by cytokine receptors [121]. JAKs then phosphorylate STAT proteins, which dimerize and translocate into the nucleus, where they regulate the expression of genes involved in cell survival and immune responses [122]. The JAK/STAT pathway plays a significant role in the immune system, but it also regulates cellular processes like hematopoiesis and the response to growth factors. Persistent activation of the JAK/STAT pathway is implicated in various cancers, particularly hematological malignancies such as leukemia, and can contribute to enhanced cell survival and uncontrolled proliferation [123,124].

The pathways discussed above—PI3K/AKT/mTOR, NF-κB, MAPK/ERK, and JAK/STAT—are not independent of each other but interact in complex networks to ensure cellular survival. For example, AKT can influence NF-κB activation, and ERK can cross-talk with the PI3K/AKT pathway [97]. This network of interactions ensures that cells respond appropriately to a wide variety of signals, maintaining homeostasis and promoting survival under both normal and stressful conditions. However, when these pathways are dysregulated, it can lead to aberrant cellular behavior, such as cancer cell growth, resistance to apoptosis, immune evasion, and metastasis [108].

### 4.2. Immune Evasion

Cervical carcinoma, often associated with high-risk human papillomavirus (HPV) infections, develops in an environment shaped by the host’s immune response. One of the hallmarks of cancer, including cervical carcinoma, is the ability to evade immune surveillance [125]. This immune evasion is a multifaceted process that involves both intrinsic changes in the tumor cells and the manipulation of the tumor microenvironment. HPV oncogenes, particularly E6 and E7, play a pivotal role by inactivating tumor suppressors like p53 and retinoblastoma protein (pRb), which not only promotes tumor growth but also dampens the host’s immune response by reducing antigen presentation through major histocompatibility complex (MHC) molecules [19].

A critical aspect of immune evasion in cervical carcinoma is the suppression of antigen presentation [20]. Tumor cells often downregulate MHC class I molecules, impairing the ability of cytotoxic T lymphocytes (CTLs) to recognize and destroy them [97]. Additionally, HPV-infected cells can secrete immunosuppressive cytokines, such as interleukin-10 (IL-10) and transforming growth factor-beta (TGF-β), which inhibit the activation and proliferation of effector immune cells while promoting regulatory T cells (Tregs) that suppress anti-tumor immunity [37].

### 4.3. Resistance to Apoptosis

A defining characteristic of cells infected with high-risk human papillomaviruses (HPV), notably HPV-16 and HPV-18, which are closely linked to cervical cancer, is their resistance to apoptosis [126]. The mechanism by which HPV evades apoptosis enables infected cells to endure and multiply despite stress or immune signals that would typically trigger programmed cell death [127]. This resistance is largely facilitated by the viral oncogenes E6 and E7, which interfere with crucial apoptotic pathways. The HPV E6 protein is pivotal in countering apoptosis by marking the tumor suppressor protein p53 for degradation via ubiquitination [128]. Under typical conditions, p53 functions as a “genome guardian,” responding to DNA damage or stress by activating pro-apoptotic genes like BAX, PUMA, and NOXA. By removing p53, E6 hinders the activation of these pathways, thus weakening the intrinsic (mitochondrial) apoptotic responses [129,130]. Additionally, E6 can directly bind with apoptotic mediators, such as Bak, to block their pro-apoptotic actions [130].

The HPV E7 protein fosters resistance to apoptosis by disabling the retinoblastoma protein (pRb) and disrupting the cell cycle. E7 breaks the link between pRb and E2F transcription factors, resulting in unchecked cell growth and avoidance of growth arrest signals [131]. This dysregulation also hinders the cell’s reaction to apoptotic signals by stimulating the production of survival genes and changing the ratio of pro- and anti-apoptotic factors. Cells infected by HPV also show resistance to extrinsic apoptotic signals, including those via death receptors [130] (e.g., Fas, TNF receptor, and TRAIL receptor). E6 can impede the activation of caspase-8, a key initiator in the extrinsic apoptotic pathway, and inhibit subsequent signaling. Furthermore, HPV infection may increase the production of anti-apoptotic proteins such as Bcl-2 and survival, boosting cell survival even in the face of immune-related apoptotic signals. HPV’s capacity to resist apoptosis is a vital factor in the ongoing survival of infected cells and their development into cancer [132]. This process not only supports the build-up of genetic mutations but also permits the evasion of immune responses, thereby playing a role in the emergence and progression of cervical cancer. Approaches aimed at counteracting the apoptotic resistance of HPV-infected cells, including the reactivation of p53 pathways or the inhibition of anti-apoptotic proteins, are being explored as prospective therapies for HPV-related cancers [133].

## 5. Conclusions

In conclusion, high-risk HPV, particularly through its E6 and E7 oncoproteins, plays a pivotal role in disrupting tumor-suppressor pathways and evading immune responses, driving the progression of cervical cancer and other HPV-related malignancies. The interplay between cytotoxic cytokines (granzymes A and B), immune cells like CTLs and NK cells, and survival pathways such as PI3K/AKT/mTOR and NF-κB underscores the complexity of the immune response and cancer biology. Understanding these mechanisms is vital for developing targeted immunotherapies and interventions that enhance immune efficacy, suppress tumor-promoting signals, and reduce HPV-related cancer burdens effectively.

## Figures and Tables

**Table 1:** Comparison of low-risk and high-risk HPV strains: prevalence and health implications.

Risk of HPV	Strain Type (Prevalence)	Implicated Diseases and Cancers	References
Low risk	6 (7%)	Genital warts Respiratory papillomatosis Giant condylomas Juvenile laryngeal or genital warts	[29,30,31]
	11 (2.5%)	Genital warts Respiratory papillomatosis	[29,31,32]
	40 (2.5%)	Cervical cancer (in rare cases) Genital warts Anal cancer (rarely) Oral cancers	[29,30,33]
	42 (5%)	Cervical dysplasia Cervical cancer (rarely) Genital warts Anal dysplasia Fingers and toes cancer	[30,31,34]
	43 (1.7%)	Cervical dysplasia Cervical cancer (rarely) Genital warts Anal dysplasia	[31]
	44 (1.7%)	Cervical dysplasia Cervical cancer (rarely) Genital warts Anal dysplasia	[35]
	54 (20.5%)	Cervical dysplasia Cervical cancer (rarely) Genital warts Anal dysplasia	[29,30]
	61 (1.4%)	Cervical dysplasia Cervical cancer (rarely) Genital warts Anal dysplasia	[33,35]
	70 (1.43%)	Cervical dysplasia Cervical cancer (rarely) Genital warts Anal dysplasia	[35]
High risk	16 (39%)	Cervical cancer Anal Oral Head Neck cancers	[36]
	18 (5.9%)	Cervical cancer Genital warts Oral, anal, penile cancers	[36]
	31 (21%)	Genital warts, some cancers (head, neck, skin)	[37]
	33 (1.6%)	Cancers (Head and neck, oral, skin, anal, penile)	[37]
	35 (7%)	Cancers (Head and neck, oral, skin, anal, penile)	[37]
	39 (9%)	Cancers (Head and neck, oral, skin, anal, penile)	[37]
	45 (10.7%)	Cancers (Head and neck, oral, skin, anal, penile)	[37]
	51 (13%)	Cancers (Head and neck, oral, skin, anal, penile)	[37]
	52 (15%)	Cancers (Head and neck, oral, skin, anal, penile)	[37]
	53 (3.6%)	Cancers (Head and neck, oral, skin, anal, penile)	[37]
	56 (6%)	Cancers (Head and neck, oral, skin, anal, penile)	[37]
	58 (6%)	Cancers (Head and neck, oral, skin, anal, penile)	[37]
	59 (9%)	Cancers (Head and neck, oral, skin, anal, penile)	[37]
	66 (10%)	Cancers (Head and neck, oral, skin, anal, penile)	[37]
	68 (12.8%)	Cancers (Head and neck, oral, skin, anal, penile)	[37]
	73 (1.6%)	Cancers (Head and neck, oral, skin, anal, penile)	[37]
	82 (4%)	Cancers (Head and neck, oral, skin, anal, penile)	[37]
	83 (2.5%)	Cancers (Head and neck, oral, skin, anal, penile)	[37]
